# Knockdown of PREX2a inhibits the malignant phenotype of glioma cells

**DOI:** 10.3892/mmr.2021.12184

**Published:** 2021-06-01

**Authors:** Liang Liu, Zhixiong Liu, Hao Wang, Long Chen, Fuqiang Ruan, Jihui Zhang, Yi Hu, Hengshan Luo, Shuai Wen

Mol Med Rep 13: 2301-2307, 2016; DOI: 10.3892/mmr.2016.4799

Following the publication of this paper, it was drawn to the Editors' attention by a concerned reader that cell invasion assay data in the article (featured in Fig. 4A) were strikingly similar to data appearing in different form in another article by different authors at different research institutions, which had already been published elsewhere at the time of the present article's submission. Furthermore, flow cytometric data featured in Fig. 2D were strikingly similar to those in another previously published paper, and cell cyle data included in Fig. 3 had apparently previously published elsewhere.

Owing to the fact that the contentious data in the above article had already appeared in different form in other articles prior to its submission to Molecular Medicine Reports, the Editor has decided that this paper should be retracted from the Journal. The authors also expressed their intention to retract the paper on the grounds that the corresponding author and several of the authors failed to confirm the approval of the final version of the manuscript. The Editor apologizes to the readership for any inconvenience caused.

